# Dynamics of *Triatoma infestans* populations in the Paraguayan Chaco: Population genetic analysis of household reinfestation following vector control

**DOI:** 10.1371/journal.pone.0263465

**Published:** 2022-02-10

**Authors:** Antonieta Rojas de Arias, Louisa Alexandra Messenger, Miriam Rolon, María Celeste Vega, Nidia Acosta, Cesia Villalba, Paula L. Marcet

**Affiliations:** 1 Centro para el Desarrollo de la Investigación Científica (CEDIC/Díaz Gill Medicina Laboratorial /FMB), Asunción, Paraguay; 2 Division of Parasitic Diseases and Malaria (DPDM), Centers for Diseases Control and Prevention (CDC), Entomology Branch, Atlanta, GA, United States of America; 3 American Society for Microbiology, NW Washington, DC, United States of America; 4 Department of Disease Control, Faculty of Infectious Tropical Diseases, London School of Hygiene and Tropical Medicine, London, United Kingdom; 5 Departamento de Medicina Tropical, Instituto de Investigaciones en Ciencias de la Salud, UNA, Asuncion, Paraguay; 6 Programa Nacional de Control de la Enfermedad de Chagas (SENEPA), Asunción, Paraguay; Fundacao Oswaldo Cruz, BRAZIL

## Abstract

**Background:**

Although domestic infestations by *Triatoma infestans* have been successfully controlled across Latin America, in areas of the Gran Chaco region, recurrent post-spraying house colonization continues to be a significant challenge, jeopardizing Chagas disease vector control and maintaining active *Trypanosoma cruzi* transmission.

**Methodology/Principal findings:**

To investigate the dynamics of triatomine reinfestation in a rural area of the Paraguayan Chaco, genetic characterization (based on 10 microsatellite loci and cytochrome B sequence polymorphisms) was performed on baseline and reinfestant *T*. *infestans* (n = 138) from four indigenous communities and adjacent sylvatic sites. House quality and basic economic activities were assessed across the four communities. Significant genetic differentiation was detected among all baseline triatomine populations. Faster reinfestation was observed in the communities with higher infestation rates pre-spraying. Baseline and reinfestant populations from the same communities were not genetically different, but two potentially distinct processes of reinfestation were evident. In Campo Largo, the reinfestant population was likely founded by domestic survivor foci, with reduced genetic diversity relative to the baseline population. However, in 12 de Junio, reinfestant bugs were likely derived from different sources, including survivors from the pre-spraying population and sympatric sylvatic bugs, indicative of gene-flow between these habitats, likely driven by high human mobility and economic activities in adjacent sylvatic areas.

**Conclusions/Significance:**

Our results demonstrate that sylvatic *T*. *infestans* threatens vector control strategies, either as a reinfestation source or by providing a temporary refuge during insecticide spraying. Passive anthropogenic importation of *T*. *infestans* and active human interactions with neighboring forested areas also played a role in recolonization. Optimization of spraying, integrated community development and close monitoring of sylvatic areas should be considered when implementing vector control activities in the Gran Chaco.

## Introduction

The Gran Chaco region, which extends through parts of Brazil, Argentina, Bolivia and Paraguay, is highly endemic for Chagas disease [1–4]. In this area, triatomine control has been implemented at the regional level through the Southern Cone Initiative, launched in 1991, with the objective of eliminating all domestic populations of *Triatoma infestans*, the vector species responsible for the majority of Chagas disease cases in Latin America [5], and controlling transfusional and congenital transmission of *Trypanosoma cruzi* [6, 7]. Despite the progress achieved through national campaigns to eliminate domestic triatomines [8], reinfestation and disease transmission persists in large parts of this region [9–11]. Control programs aim to reduce domestic populations of *T*. *infestans* by spraying human households with residual insecticides [12] but have not included any actions against sylvatic populations [10, 13–15]. The causes and determinants of triatomine reinfestation are not fully known, nor is the relative importance of invasion from sylvatic foci to the re-establishment of peridomestic or domestic bug populations. This previously unaddressed challenge is now considered a priority issue for the Southern Cone Initiative [16, 17] as increasing numbers of wild foci are reported [18].

*T*. *infestans* sylvatic populations have been reported in Argentina [19], Bolivia [13, 20–23], Chile [24, 25] and Paraguay [26]. Population genetics studies in the Bolivian Valleys have provided evidence of Chagas disease transmission risk from sylvatic populations of *T*. *infestans* that move into intra- and peridomestic areas [27, 28]. Some remnant triatomine populations present inside the houses after spray campaigns in endemic communities were found to be related to sylvatic colonies, suggesting that the sylvatic environment could be involved in the re-infestation process and may contribute to the re-establishment of populations in treated houses [29]. Furthermore, in the Chaco regions of Bolivia and Argentina, several studies have identified persistent triatomine bug populations inside houses, which were also resistant to pyrethroid and organophosphate insecticides [30–33].

In Paraguay, the Central Chaco region has had the highest rates of human *T*. *cruzi* infection and triatomine infestation, composed mainly of indigenous people belonging to different ethnic groups [34, 35]. Local indigenous houses are typically single-roomed, without peridomicile areas [36] and are directly adjacent to the dry forest. These indigenous groups live on a fragile subsistence economy; many are hunter-gatherers that often enter the forest to hunt wild animals, collect fruits and honey for consumption, as well as to prepare charcoal for sale [18, 26]. Historically, the Paraguayan National Chagas Disease Control Program has performed several household pyrethroid insecticide spray campaigns in the region. However, rapid domestic reinfestation was observed despite the efforts of the Program [37, 38]. In 2003, the National Chagas Disease Control Program estimated an average pre-sprayed triatomine infestation rate of 17%, reaching up to 45% in some localities of the Central Chaco. In 2005, the prevalence of re-infestation ranged from 5 to 10% in some communities.

The main objectives of this work were to identify the putative sources of reinfestant bugs after a mass spray campaign in a group of rural communities in the Paraguayan Chaco, and to assess the role of sylvatic populations of *T*. *infestans* in the reinfestation process. For this purpose, domestic and peridomestic populations before and after community-wide insecticide spraying of houses were compared using high-resolution molecular markers. The genotypes of sylvatic *T*. *infestans* populations captured in the area were also analyzed to determine whether gene-flow occurs between sylvatic and domestic populations. Study findings were interpreted in the wider context of ongoing anthropogenic processes and additional environmental factors which act together to influence triatomine reinfestation dynamics.

## Materials and methods

### Study site characteristics

**Fig 1:**
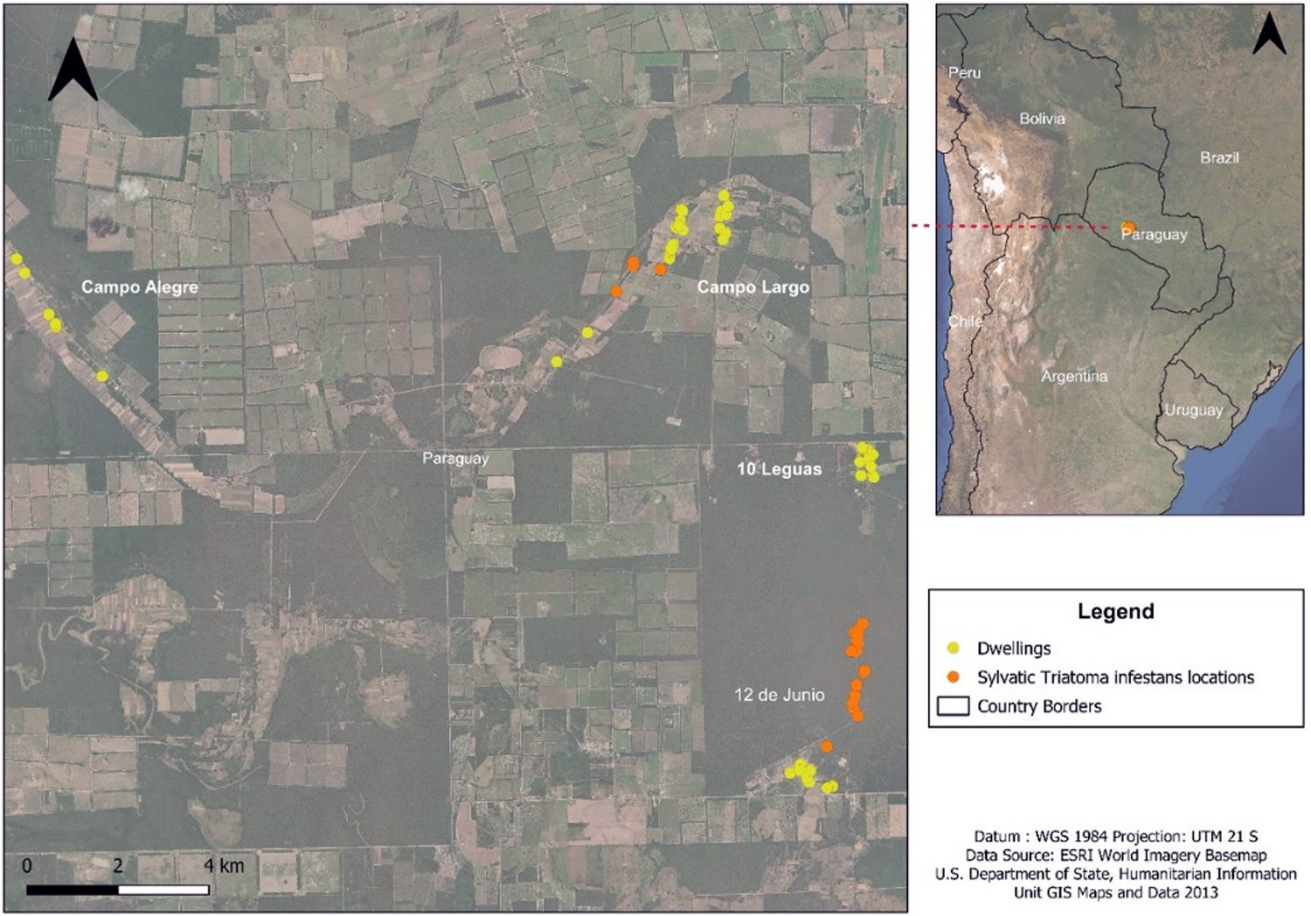
Map of the study area indicating positions of the four communities: Campo Alegre (Mariscal Estigarribia, Boquerón Department) and Campo Largo, 10 Leguas and 12 de Junio (Benjamín Aceval, Presidente Hayes Department). Yellow and orange dots indicate triatomine capture sites inside houses or from the sylvatic environment [26], respectively. Note: multiple bugs were collected per capture site. Map was made using ESRI 2016. "World Imagery" [basemap]. Scale Not Given. "World Imagery Map". October 13, 2021. https://www.arcgis.com/home/item.html?id=10df2279f9684e4a9f6a7f08febac2a9. (Oct 26, 2021). QGIS Development Team, 2021. QGIS Version 3.20.3. Geographic Information System. Open Source Geospatial Foundation. Data source: DGEEC 2012. https://www.ine.gov.py/microdatos/microdatos.php. Detailed World Polygons (LSIB), South America, 2013. http://purl.stanford.edu/vc965bq8111.

The study area has been described in detail in previous publications [18, 26]. Briefly, this study was carried out in an area distributed over approximately 500 km^2^ and located 634 km from Asunción in the Central Paraguayan Chaco (Fig 1), a xeromorphic forest of the Gran Chaco ecoregion, characterized by extreme temperatures that reach 45°C during the summer and 7°C in the wintertime [18]. The study area contained four communities (Campo Alegre, CA: -22.4944, -60.0314; Campo Largo, CL: -22.49432, -59.54476; 10 Leguas, 10L: -22.52244, -59.52186; and 12 de Junio, 12J: -22.56137, -59.52572) with an estimated population of 1,422 inhabitants, chosen because they were contiguous villages with high rates of *T*. *infestans* infestation and high prevalence of *T*. *cruzi* infection. A characteristic of these indigenous communities was the formation of satellite colonies, consisting of a small number of dwellings, which were generally isolated from older localities. Within these communities, human migration is common amongst residents because many are direct relatives of each other, or in search of water sources during times of drought. In this context, passive transport of triatomines between villages is likely to occur, as people travel with bags of clothes and even some construction materials used in their previous households (e.g. wood and roof parts) [18].

The communities of 10L and 12J are composed of the Angaité ethnic group that belong to the Maskoy linguistic family (registered linguistically as Enlhet-enenlhet) and are part of one of the six ethnic groups of this family. CL belongs to the Enlhet ethnic group of the same family. These ethnic groups are indigenous to the south-eastern region of the Paraguayan Chaco. It is believed that these communities came from Puerto Casado, an area located 300 km away from 10L some decades ago and still actively travel back and forth to Puerto Casado. In contrast, CA is composed of the Nivaclé or Chulupí ethnic group that belongs to the linguistic family Mataco Mataguayo. This ethnic group is distributed from the center of the Chaco to the Pilcomayo river and currently inhabits the central-eastern part of the Paraguayan Chaco (a more detailed description of study communities can be found in S1 File).

**Fig 2:**
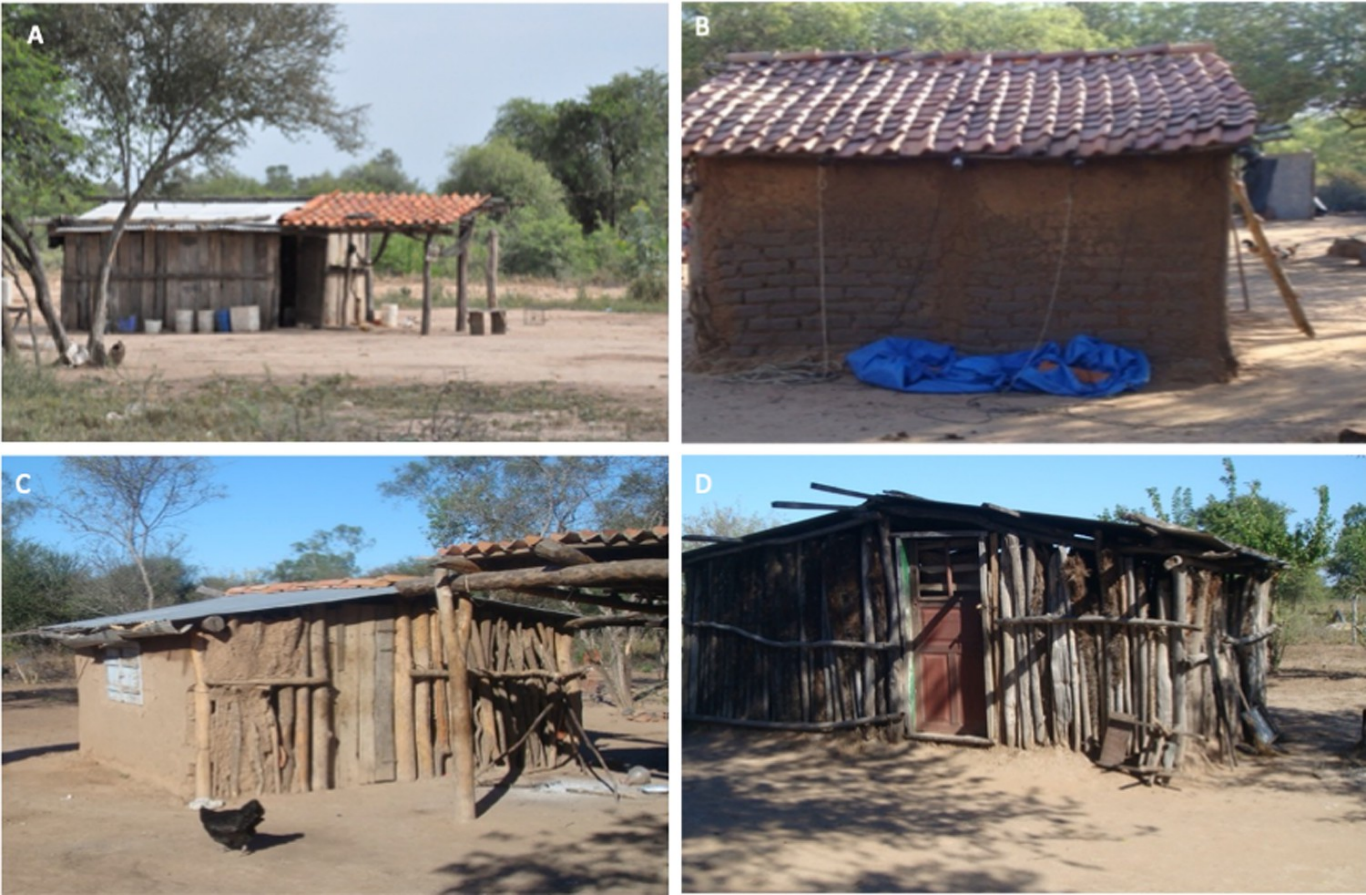
Examples of typical houses, constructed with local materials in Campo Alegre (A), Campo Largo (B), 10 Leguas (C) and 12 de Junio (D).

The four study communities are separated by 5 to 15 km (Fig 1) and are inter-connected by paved roads. Administratively, 12J, 10L and CL are located in the rural district of Benjamín Aceval (Presidente Hayes Department), while CA is in the district of Mariscal Estigarribia (Boquerón Department). Within these communities, most inhabitants lived in houses with dirt floors, with walls constructed from tree logs and un-plastered mud-bricks, “french walls” (wall structure of wooden sticks filled with clay and mud), or mixtures of both types. Roofs were mostly constructed of zinc. Communities collected water from the roofs through gutters connected to water-storage containers. While inhabitants had access to similar local building materials, there were distinct differences in house construction between communities (Fig 2).

### Assessing house construction

In the framework of a socio-economic survey carried out previously in the four study communities, the quality of construction materials of 270 houses was evaluated according to the CALMAT quality index used by the National Institute of Statistics and Censuses (INDEC) of Argentina [39]. The methodology used has been explained elsewhere [36].

### Household entomological surveys and spraying

The baseline survey, carried out from March-August 2008 in the four communities, included searching for triatomine bugs in domestic and peridomestic structures in all houses (n = 325). The “man hour method” was followed: two trained collectors searched for triatomine bugs in bedroom areas for 30 minutes per house and for 15 minutes in the peridomicile (if present).

In August 2008, a residual spraying campaign with pyrethroid insecticide was carried out in the whole area. Houses and peridomiciles (if present) were sprayed with lambda-cyhalothrin (ICON^®^ 10% wettable powder) at 30 mg/m^2^ using manual compression sprayers (Hudson^®^). Our team accompanied the spraying brigades and searched each house for triatomines immediately after spraying (baseline collections). Captured bugs were placed in plastic containers, identified with the house number, name of the household owner and the specific collection site. A self-sealing plastic bag was given to each family to place any triatomine bugs captured in the domestic or peridomestic areas after spraying (immediately and at 1, 3, 6, 9 and 12 months later).

Sylvatic bugs were captured using a trained dog, as previously described [26]. Sylvatic locations where bugs were found were georeferenced and are shown in Fig 1. Searches were carried out five times over four months (May-August 2010) during the post-spraying period [26]. Sylvatic searches were performed in adjacent areas to all communities but triatomines were only found around 12J and CL. The average distance from the domestic structures to sylvatic capture sites ranged from 230 m to 3.6 km.

Bugs captured immediately after spraying (knock-down) were pooled per locality and were considered the baseline population. Bugs captured over 12 months post-spraying (at 1, 3, 6, 9 and 12 months) were pooled per locality and were considered the “reinfestant” population. Henceforth, this group refers to the entire repopulation process, irrespective of triatomine source, i.e. individuals may be derived from external sources (e.g. from the sylvatic or peridomestic environments) or internal colonization (e.g. triatomines which escaped chemical control in the house and had subsequently propagated). Bugs captured over four months post-spraying from the sylvatic environment surrounding each community were pooled per locality and were considered the “sylvatic” population.

### Detection and isolation of *Trypanosoma cruzi* in triatomines

*Trypanosoma cruzi* infection of *T*. *infestans* and *T*. *sordida* captured alive was determined by microscopic observation at 400X of a drop of feces diluted in saline solution. Feces from positive domestic *T*. *infestans*, collected during the baseline survey were cultured for genotyping of *T*. *cruzi* discrete typing units (DTUs) [40–42]; parasite DNA was extracted using DNeasy kits (Qiagen, USA). Genetic characterization of DTUs was undertaken using a combination of amplicon profiles from PCR reactions targeting the D7 divergent domain of the 24Sα rRNA region [43], the size variable domain of the 18S rRNA region [44], the non-transcribed spacer of the mini-exon gene [43] and a PCR–restriction fragment length polymorphism (PCR–RFLP) of the intergenic region of heat shock protein 60 [42].

### Multilocus microsatellite genotyping

*T*. *infestans* specimens for molecular analysis were randomly selected to represent “baseline”, “reinfestant” (from each of the four study communities) and “sylvatic” populations. In sites where low numbers of *T*. *infestans* were collected, all individuals were included for analysis, e.g. CL-sil. DNA extraction was carried out using four legs from each of 138 *T*. *infestans* specimens, ground to a fine powder with individual grinders (Kontex) in the presence of liquid nitrogen, incubated overnight at 37°C in 1 ml lysis buffer, and processed with a standard sequential phenol-chloroform protocol [45, 46]. Multilocus genotypes (MLGs) for 10 microsatellite loci (Tinf_ms3, Tinf_ms5, Tinf_ms19, Tinf_ms22, Tinf_ms23, Tinf_ms27, Tinf_ms42, Tinf_ms56, Tinf_ms64 and Tinf_ms65) were generated using genotyping conditions, as previously described [47, 48].

### Microsatellite analysis

DNA fragment detection with 1 bp resolution was performed with an automated DNA sequencer (ABI 3130, Applied Biosystems) and size determination was obtained with GeneMapper 4.1 (Applied Biosystems). Allele number per locus and population was obtained directly after binning, and mean allele number among all loci were compared. The inbreeding coefficient Fis [49]; allelic richness Ar [50], gene diversity or expected heterozygosity (He) and observed heterozygosity (Ho) [51] were obtained with the Excel add-in MS_tools.xla for Microsoft Excel or with FSTAT2.9.3.2 [52]. Bonferroni correction was applied to determine p-values for multiple comparisons [53]. The fit to Hardy-Weinberg (HW) expectations was evaluated using the U score test available in Genepop 4.2, under the null hypothesis of random union of gametes. Sample-size-corrected private (population-specific) allele frequency per locus (PA/L) was calculated in HP-RARE [54]. Pairwise *F*_ST_ values and significance levels were calculated with ARLEQUIN 3.01 [49]. Fisher’s exact test for population differentiation comparing genic and genotypic frequencies was implemented in Genepop 4.2. Population clustering was explored using a neighbor-joining (NJ) tree based on pairwise distances (*D*_AS_: 1-proportion of shared alleles at all loci/n). A Mantel’s test for the effect of isolation by distance within populations (pairwise genetic vs. geographic distance) was performed in GenAlEx 6.5 using 10,000 random permutations [55].

### Population structure

Population genetic structure was examined using a Bayesian model-based approach [56–58] implemented in STRUCTURE 2.3.4. Each individual MLG in the sample was probabilistically assigned to one of *K* populations, or jointly to two or more populations if their genotypes may have had an admixed origin. Simultaneously, the method determined the number of significant *K* genetic clusters within the total sample. The number of clusters evaluated (= *K*) ranged from 1 to 10. The analysis was performed using 35 replicate runs per *K* value, a burn-in period length of 50,000 and a run length of 50,000. The analysis model used was the admixed and correlated allele frequencies, with no prior information on the origin of the individuals. The final selection of the sample *K* value was based on the log-probability of the data between successive *K* values [59], implemented in the online version of STRUCTURE HARVESTER [60].

### Mitochondrial genotyping

The mitochondrial cytochrome B (cytB) gene was targeted for amplification as described in Lyman et al. 1999 [61], to produce a 415 bp fragment with no insertions or deletions. PCR amplification products were visualized in 2% agarose gels, stained with ethidium bromide, and successful amplicons were purified for cycle sequencing using a MultiScreen®HTS Vacuum Manifold (Millipore, USA). DNA cycle sequencing reactions were performed for both strands with a BigDye 3.1 sequencing kit (ThermoFisher), following standard manufacturer protocols, and sequenced in an automated DNA sequencer (ABI 3130, Applied Biosystems). Sequences were manually assembled and aligned with SeqmanPro (DNASTAR, Inc) and BioEdit 7.2.0 [62].

### Mitochondrial analysis

Standard genetic variability (haplotype diversity, Hd and nucleotide diversity, π) and differentiation among sequences were evaluated using DnaSP version 5.1 software [63]. To investigate the possible directionality and history of gene flow among populations, we compared the sequences obtained in this work with previously reported *T*. *infestans* haplotypes, deposited in GenBank (S2 File). Sequences were trimmed from 415bp to 388bp and a Nexus matrix was constructed for haplotype network analysis in PopART using a median-joining model based on 1000 iterations with default parameters [64, 65].

### Ethical approval

The study was approved by the local communities through their leaders and school teachers. The informed consent was read by the school teacher in each locality and signed on behalf of the community after a collective meeting was held with each leader. During the entomological surveys, all participants were present at the time of the household evaluation and gave verbal approval prior to house inspection; all participants had the right to withdraw from the study at any point with impunity. The Ethics Committee of the Moisés Bertoni Foundation (IDRC grant # 103696–009, revision 07/27/2007) approved the project from February 2008 to March 2010.

## Results

### House construction and condition

Among the dwellings evaluated for construction quality, 83% (224/270) consisted of just one room (excluding latrines and kitchens) and usually inhabitants slept outside. Most householders (83%) prepared their food outdoors and the bathroom was generally outside the house (98.5%) [36]. Only 9.3% of houses (25/270) had structures other than the kitchen and latrines in the peridomicile, e.g. chicken coops, vegetable gardens or other animal corrals.

**Fig 3:**
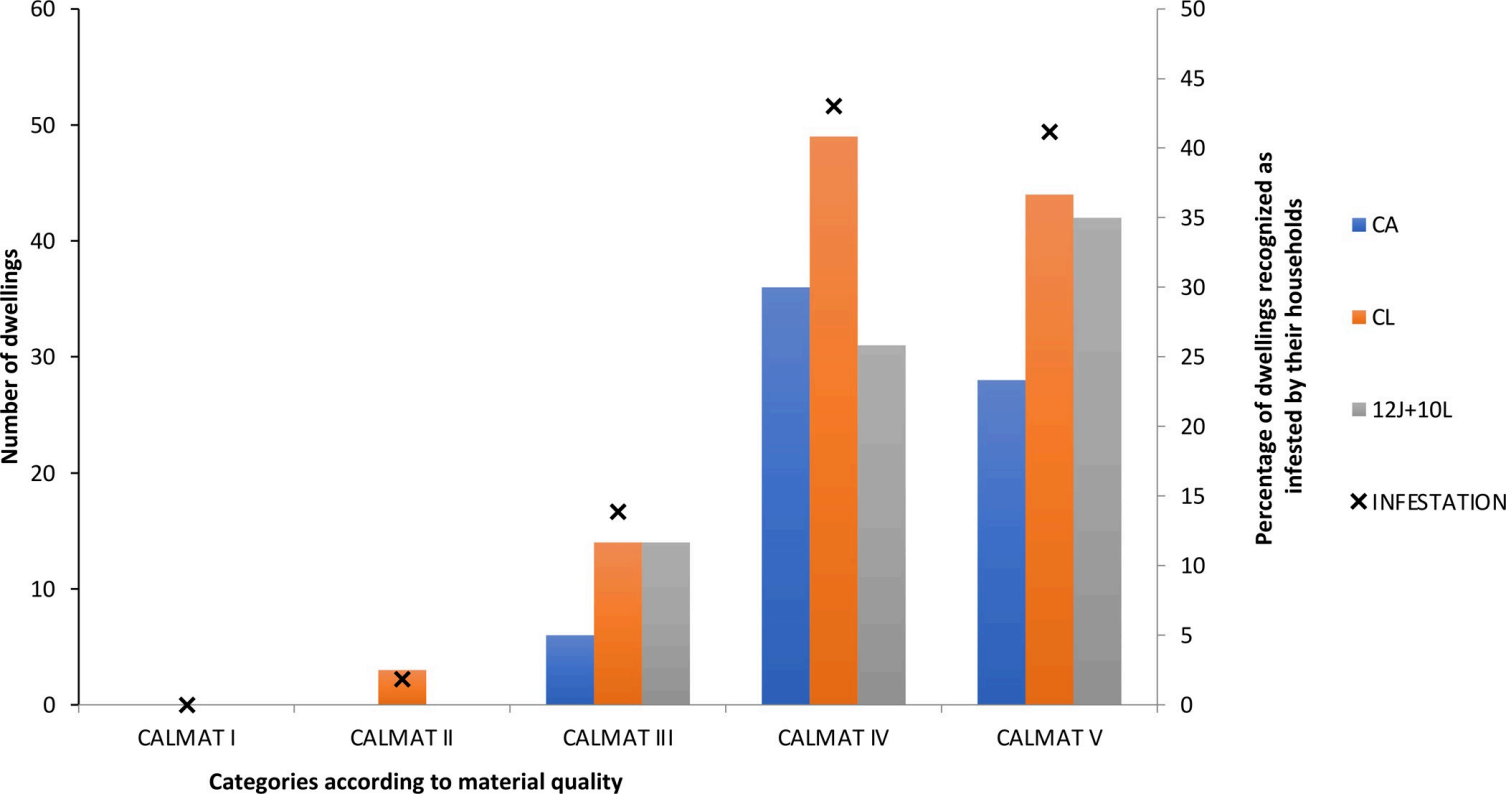
Quality of housing construction materials used in dwellings in the four study communities (CA, CL, 12J and 10L). CALMAT I: The dwelling contains durable resistant materials and solids for all parameters (floors, walls, ceilings) and incorporates all elements of insulation and plastering. CALMAT II: The dwelling presents durable materials and solids in all components, but it lacks elements of insulation or plastering in at least one of its components (floors, walls, and ceilings). CALMAT III: The dwelling presents durable materials and solids in all parameters, but it lacks elements of insulation or plastering in all its components, or either has sheet metal or fiber cement roofs or no ceiling; or walls of sheet metal or fiber cement. CALMAT IV: The dwelling presents materials not durable or solid in at least one of the components, but not in all of them. CALMAT V: The dwelling has no resistant or solid materials in any components [39].

The low quality of building materials used to construct the houses rendered them highly vulnerable to triatomine colonization and reinfestation, and a direct correlation between worsening CALMAT category and level of household infestation was observed (Fig 3). In the communities of the Nivaclé and Enlhet ethnic groups (CA and CL, respectively), the quality of the materials used was concentrated in the CALMAT IV and V categories; CALMAT III and II categories were also found in the latter ethnic group (Fig 3). In the Angaité communities (10L and 12J), the majority of houses were assigned to the poorest quality CALMAT categories (Fig 3).

### Baseline and post-spraying entomological surveys

A total of 986 triatomines, including 880 (89.2%) *T*. *infestans* and 106 (10.8%) *T*. *sordida*, were captured in the baseline survey carried out in 325 dwellings, representing an average area-wide indoor *T*. *infestans* infestation rate of 36.3% (118/325). Infestation levels were twice as high in 12J (70%) than in the other communities (25–30%). Area-wide peridomestic infestation was 2.8% (9/325), although no *T*. *infestans* were collected from peridomestic areas during the baseline survey (all were *T*. *sordida* sampled from chicken coops; *T*. *sordida* specimens were not included in subsequent molecular analyses). The level of indoor colonization (percentage of houses with nymphs) by *T*. *infestans* in the baseline assessment ranged from 55 to 71% (Table 1A).

**Table 1:** 

Community	No. houses inspected	No. houses infested indoors (%)	No. houses with infested peridomestic sites* (%)	Indoor colonization rate** (%)	No. triatomines collected (*T*. *infestans*)	No. *T*. *infestans* collected alive (%)	No. *T*. *cruzi* infected *T*. *infestans* (%)
**A:** Total triatomine infestation after knockdown and manual collection during the baseline survey, March-August 2008.							
12 de Junio	60	42 (70)	0 (0)	30 (71.4)	519 (517)	404	46 (11.4)
10 Leguas	56	17 (30)	1 (1.8)	10 (58.8)	125 (124)	94	2 (2.1)
Campo Largo	129	39 (30.2)	3 (2.3)	23 (58.9)	236 (178)	149	8 (5.4)
Campo Alegre	80	20 (25)	5 (6.3)	11 (55)	106 (61)	21	5 (23.8)
**Total**	**325**	**118 (36.0)**	**9 (2.8)**	**74 (62.7)**	**986 (880)**	**668 (75.9)**	**61 (9.1)**
**B:** Total triatomine reinfestation after knockdown and manual collection during 12 months post-spraying (2008–2009).							
12 de Junio	61	6 (9.8)	4 (6.5)	2 (33.3)	82 (78)	73	3 (4.1)
10 Leguas	56	0	1 (1.8)	0	1 (0)	0	NA
Campo Largo	132	12 (9.1)	10 (7.6)	7 (58.3)	33 (24)	18	0
Campo Alegre	84	1 (1.2)	3 (3.6)	0	19 (4)	3	2 (66.6)
**Total**	**333**	**19 (5.7)**	**18 (5.4)**	**9 (47.4)**	**135 (106)**	**94 (88.7)**	**5 (5.3)**

*All triatomines captured in peridomestic sites at baseline were *T*. *sordida*. Post-spraying, all triatomines captured in peridomestic sites were *T*. *infestans*. NA: Not applicable.

**Colonization rate refers to the presence of nymphs indoors, defined as the number of houses with *T*. *infestans* nymphs out of total infested houses.

During the 12-month post-spraying entomological monitoring, persistent indoor *T*. *infestans* infestation was observed in 12J and CL whereas no infestations were observed in 10L, and *T*. *infestans* was found indoors in only one household from CA (Table 1B). The *T*. *infestans* infestation rate dropped sharply in 12J from 70% to zero in the first month post-spraying but began to rise three months later (4.9%; 3/61), reaching 9.8% (6/61) by the end of the post-intervention year. In CL, infestation by *T*. *infestans* decreased from 30.2% at baseline to 9.1% (12/132) after spraying. In the first four months post-spraying, a total of 22 and 5 *T*. *infestans* were collected from the sylvatic environment surrounding 12J and CL, respectively [26].

It is noteworthy that infestations of peridomestic areas in CL increased from 2.3% to 7.6% following spraying, likely due to the installation of chicken coops one month before the entomological monitoring. Although the triatomines captured in peridomestic structures were all *T*. *sordida* during the baseline survey, 10 *T*. *infestans* were found in peridomestic areas post-spraying and no *T*. *sordida* were collected. In 12J, there was no peridomestic infestation at baseline, however, *T*. *infestans* were found in 6.6% (4/61) of peridomestic sites post-spraying.

In total 668 *T*. *infestans* and 78 *T*. *sordida* were examined for the presence of *T*. *cruzi*, with 9.1% and 0% of triatomines infected, respectively. *T*. *infestans* captured in CA presented the highest *T*. *cruzi* prevalence pre-spraying (23.8%; 5/21) which remained remarkably high post-spraying (66.6%; 2/3), although only 3 live *T*. *infestans* were captured after spraying. In 12J, parasite prevalence fell from 11.4% (46/404) pre-spraying to 4.1% (3/73) post-spraying. No live infected *T*. *infestans* were captured post-spraying in CL or 10L, where pre-spraying prevalences were 5.4% (8/149) and 2.1% (2/94), respectively. Furthermore, none of the sylvatic *T*. *infestans* collected in 12J and CL were infected.

### *Trypanosoma cruzi* genotypes in the study area

*T*. *cruzi* parasites were successfully isolated and cultured from 80.3% (49/61) of infected *T*. *infestans*, sampled at baseline. The most common *T*. *cruzi* DTUs infecting domestic *T*. *infestans* were TcV (51.0%; 25/49), followed by TcVI (46.9%; 23/49), and one of their parental lineages TcII (2.0%; 1/49) (Table 2). No mixed DTU infections were observed in any individual bugs.

**Table 2:** Distribution of *T*. *cruzi* discrete typing units (DTUs) identified in study communities during the baseline entomological survey.

Localities	Total number of *T*. *infestans* infected	Total number of parasites isolates*	DTUs		
			TcII	TcV	TcVI
**12 de Junio**	46	37	-	16	21
**10 Leguas**	2	2	-	-	2
**Campo Largo**	8	7	1	6	-
**Campo Alegre**	5	3	-	3	-
**Total**	61	49	1	25	23

*Twelve parasite isolates were contaminated with bacteria/fungus during the process of isolation and culturing from triatomine feces and therefore were not included in further analysis.

### Genotyped triatomine population characteristics

A total of 138 *T*. *infestans* (from “baseline” = bas; “reinfestant” = re and “sylvatic” = sil populations) were genotyped across 10 polymorphic microsatellite loci, each presenting a unique MLG. All populations demonstrated uniform levels of genetic diversity, as evidenced by similar measurements of average gene diversity (He) (range of 0.54–0.74), allelic richness (range of 2.1–2.78) and private alleles per locus (range of 0.16–0.59) (Table 3). The allele frequencies of all but two populations met HW expectations of random mating within populations. The populations that did not present HW (10L-bas and CL-re) showed significant heterozygote deficits (Table 3).

**Table 3:** Population diversity parameters.

Population	Sample size	He ± SD	Ho	PA/L ± SD	av#A	avPIC values	av Ar	Fis (W&C)
12J-bas	24	0.65 ± 0.04	0.58	0.34 ± 0.01	6.1	0.59	2.48	0.106
12J-re	25	0.69 ± 0.04	0.55	0.43 ± 0.04	7.0	0.63	2.59	0.208
12J-sil	22	0.68 ± 0.03	0.60	0.44 ± 0.07	6.7	0.63	2.60	0.116
10L-bas	18	0.54 ± 0.05	0.51	0.23 ± 0.03	4.2	0.47	2.15	0.061**
CL-bas	16	0.74 ± 0.04	0.72	0.59 ± 0.07	6.8	0.68	2.78	0.026
CL-re	15	0.64 ± 0.05	0.63	0.18 ± 0.008	4.8	0.57	2.43	0.012**
CL-sil	5	0.71 ± 0.03	0.59	0.16 ± 0.42	3.7	0.56	2.60	0.197
CA-bas	13	0.7 ± 0.04	0.51	0.57 ± 0.23	5.8	0.62	2.62	0.280
Total	138							

**Bas:** baseline; **re:** reinfestant; **sil:** sylvatic; **He:** expected heterozygosity = average gene diversity [51; **Ho:** observed heterozygosity; **PA/L**: private allele per locus; **av#A:** average number of alleles per population; **avPIC:** averaged Polymorphism Information Content [66]; **av *A***_**r:**_ Averaged allele richness [50]; **Fis:** inbreeding coefficient [49]; ** significant heterozygote deficit p>0.05.

Pairwise Fisher exact tests for genic and genotypic differentiation averaged across all loci did not detect significant differentiation for the following populations pairs after applying the Bonferroni correction for multiple comparisons (p>0.00009): 12Jbas, -re or -sil; CL-bass and -re; CL-sil with neither 12J population.

*F*_ST_ pairwise distance among populations ranged between 0.006–0.128. No significant differentiation was detected among the 12J baseline and reinfestant populations, the reinfestant and sylvatic populations from 12J, the baseline and reinfestant populations from CL and all pairs compared to CL-sil (Table 4); the latter observation is likely due to the small population sample size of CL-sil (n = 5).

**Table 4:** *F*_ST_ values per community population pairwise comparisons.

	12J-bas	12J-re	12J-sil	10L-bas	CA-bas	CL-bas	CL-re	CL-sil
12J-bas	-	0.025	0.018*	0.084*	0.072*	0.058*	0.074*	0.027
12J-re		-	0.023	0.08*	0.062*	0.049*	0.06*	0.006
12J-sil			-	0.114*	0.057*	0.081*	0.09*	0.008
10L-bas				-	0.128*	0.102*	0.133*	0.126
CA-bas					-	0.070*	0.104*	0.026
CL-bas						-	0.011	0.054
CL-re							-	0.111

* = Significant after Bonferroni correction. Indicative adjusted nominal level (5%) for multiple comparisons p<0.0018.

To determine the extent of spatial genetic structure (isolation by distance), a Mantel’s test was conducted and no significant isolation by distance was observed between populations (R_XY_ = 0.322; *P* = 0.11).

**Fig 4:**
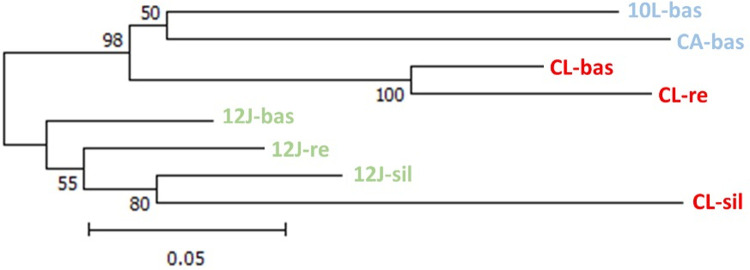
NJ tree based on Da pairwise distance values (estimated in POPTREEW) based on MLGs of 10 microsatellite loci.

Population pairwise genetic distances, evaluated using the NJ algorithm, indicated that the most significant and clear clustering was between the baseline and reinfestant populations from CL, grouped with a bootstrap support of 100 (Fig 4). A second level clustering was represented by two groups, one including CL-bas, CL-re, CA-bass and 10L-bas (bootstrap support of 98), and the other group comprising the three populations from 12J and the CL-sil population, with the two sylvatic populations clustering closer together than the rest (Fig 4).

**Fig 5:**
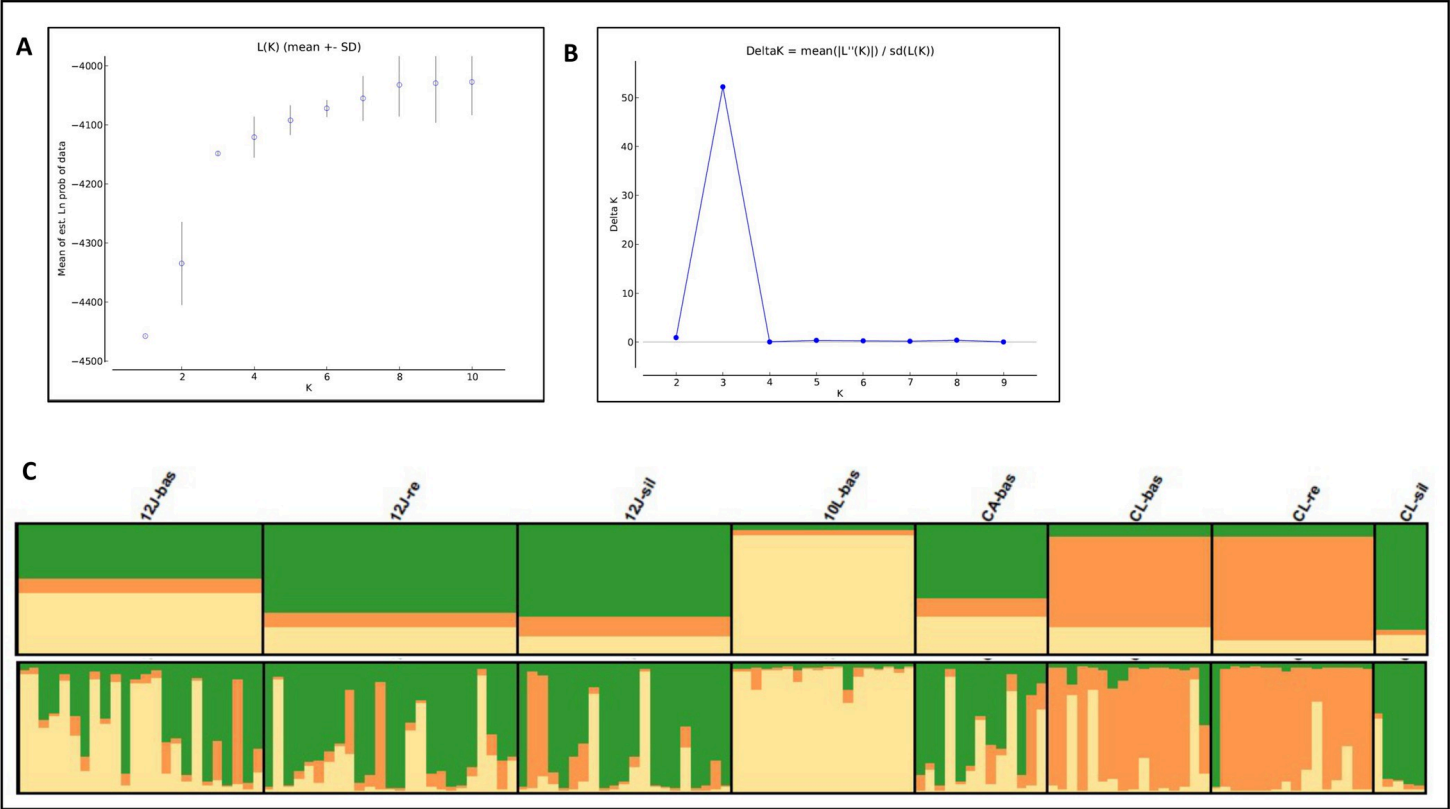
Bayesian cluster analysis results of *T*. *infestans* populations from domestic and sylvatic areas from four communities from Paraguay. 5A) Mean of estimated Ln likelihood of the probability data adjust to the model with K clusters (Ln(P)), versus K number of genetic clusters considered [58]. 5B) Graphic representation of Delta K = mean ((|L″(K)|)/SD(L(K)) [59], to evaluate the rate of change in the Ln(P) versus the number of genetic clusters considered (*K =* 1–10). 5C) Bar plot representing the *a priori* assignment probability of each multilocus genotype (MLG) to be assigned to a specific genetic cluster (represented by colors). Upper: graphic representation of the population relative composition to each genetic cluster (K = 3). Lower: each vertical bar represents an individual and the different colors are the probability with which each individual resulted assigned to each of three genetic clusters.

A Bayesian based assignment of individual genotypes determined an optimal number of three genetic clusters to describe the dataset (Fig 5). At this clustering level, the mean log posterior probability (Ln(PD)) reached a plateau indicating significant individual assignment to a single cluster above 81% [58, 67].

The graphic representation of the assignment results reflects the individual and population probabilities of belonging to a specific genetic cluster. Briefly, different degrees of genetic divergency within populations were observed. The domestic population from 10L was the only genetically homogeneous (i.e. composed of individuals belonging to the same genetic cluster (Fig 5C) whereas the remaining populations were comprised of individuals from different genetic origins (i.e. individuals assigned to any of the three genetic clusters (different colors) albeit in substantially different proportions. Considering the genetic composition (frequency of individuals assigned to a given genetic cluster), populations from 10L-bas and CL-bas and -res were clearly distinguishable from the rest.

### Mitochondrial phylogeographic analysis

A fragment of the cytochrome B gene (cytB) was sequenced from 124 out of 138 *T*. *infestans* from all populations (12J-bas = 24, 12J-re = 26, 12J-sil = 21, 10L = 18, CA-bas = 12, CL-bas = 11, CL-re = 9 and CL-sil = 3) and assembled into a 415bp alignment. A total of four haplotypes (haplos a-d) were observed, determined by 7 variable sites, including two heteroplasmid sequences (haplos a/b and b/c in individuals from 12J-re and CA-bas, respectively). All haplotypes were submitted and are accessible in GenBank (accession numbers: haplo a—KY654077; haplo b—KY654076; haplo c—KY654080; and haplo d—KY654078).

**Fig 6:**
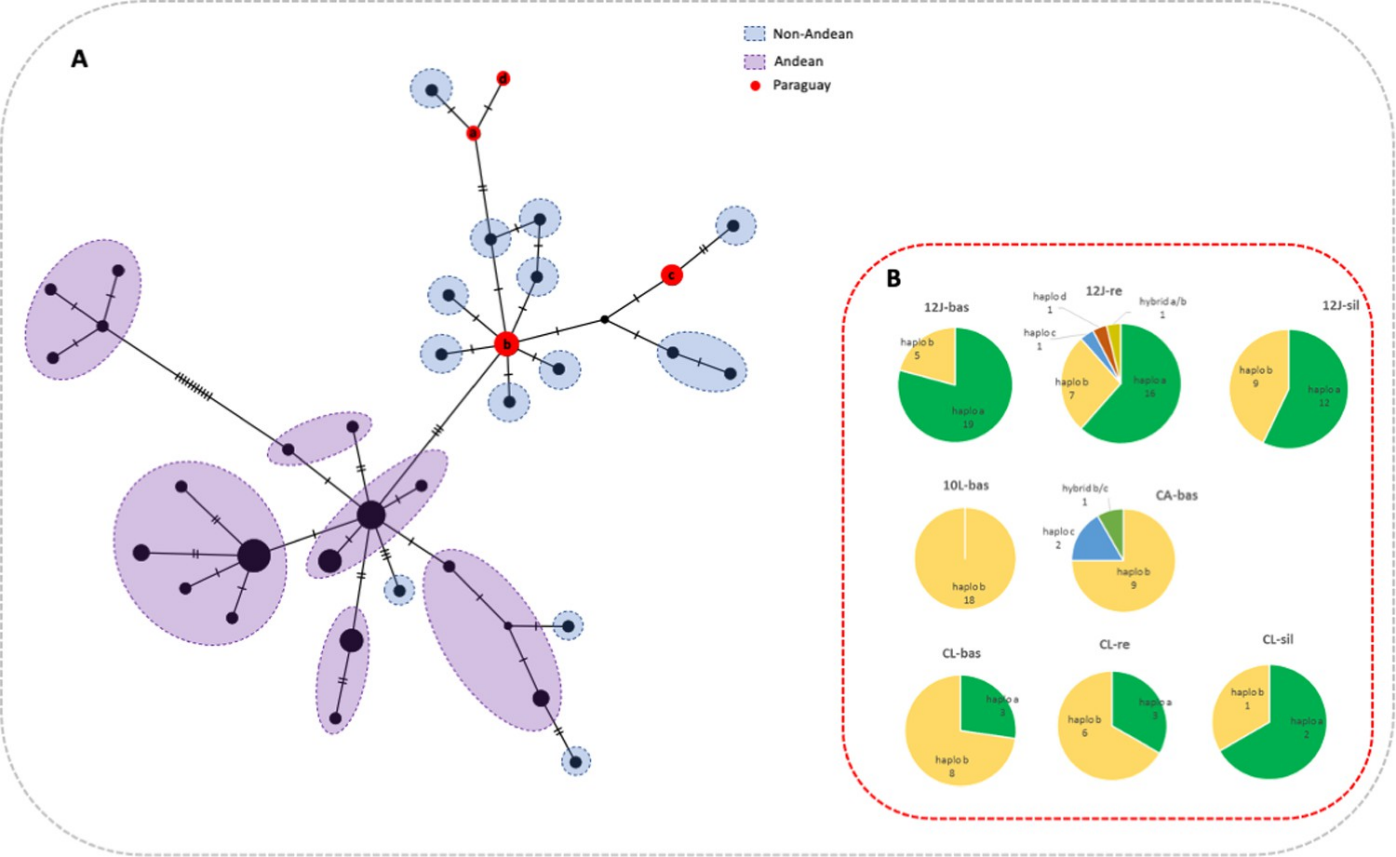
**A:** Haplotype network based on a median-joining model, constructed from sequences identified in this study (shown in red) and 71 additional *T*. *infestans* CytB haplotypes available in Genebank from Argentina and Bolivia [69–74]. Sequences were classified as ‘Andean’ or ‘Non-Andean’ based on geographical origin (S2 File). The distance among haplotypes is represented by the number of mutational steps between them. Note: that several haplotypes converged into a few centrally located haplotypes as the original sequence length was shortened for this analysis, causing some variable sites to be lost. **B:** Cytochrome B haplotype distribution among study communities. Numbers indicate individuals with that haplotype detected per community. Hybrid haplotypes are heteroplasmid individuals.

Two haplotypes (a and b) accounted for 96% (121/126) of individuals and were prevalent in variable proportions among populations (Fig 6A). The most genetically diverse population was 12J-re, where all four CytB haplotypes were identified (Fig 6A). Haplotype a was entirely absent from 10L and CA.

Haplotype b was first reported in populations from Argentina, Bolivia and Brazil (CytBHapD; alternate GenBank accession numbers: HQ333218.1) [68] and since then multiple studies have shown that this haplotype is widely distributed across the region and has been detected in high frequencies in several studies [69]. Haplotype a has not yet been reported in any other area outside our study communities in Paraguay and was first identified in a previous publication from our group [26].

In order to interpret the results of this study in the context of known phylogeographic patterns of *T*. *infestans* populations across the Gran Chaco region, we built a haplotype network with 71 previously reported CytB haplotypes for *T*. *infestans* from Argentina and Bolivia [69–74] (S2 File and Fig 6B). Haplotypes from this study were trimmed to 388bp to align with the majority of previously reported sequences. This network analysis indicated that haplotype b (reported in this study and others) was most likely ancestral, occupying a central node from which several derivatives have emerged and spread across the endemic region. By comparison haplotype a has likely evolved by secondary diversification and thus far has only been reported in the study area in Paraguay.

## Discussion

Given the absence of highly efficacious chemotherapy to treat adult Chagas disease patients [75], the lack of chemoprevention or a vaccine, coupled with barriers of poverty preventing access to early diagnosis and treatment [76], vector control is absolutely critical to prevent new *T*. *cruzi* infections in endemic regions of the Gran Chaco, where indices of household triatomine infestations remain high [77, 78]. In these areas, the determinants of house reinfestation after spraying are complex, involving the interplay of a number of factors: the effective population size of pre-spraying household infestations [11], the presence and proximity of sylvatic foci [26–28], the dispersal capacity of *T*. *infestans* [79, 80], triatomine nutritional status [80] and availability of blood meals in the peridomicile [78, 81], poor housing quality and associated socio-economic status [9, 11, 77], the spread of pyrethroid resistance in *T*. *infestans* [82, 83], as well as reported reduced efficacy of pyrethroid insecticides in peridomestic environments [81]. In particular, differentiating between reinfestation resulting from residual individuals within the house that survived insecticidal treatment, or house invasion/from peridomestic areas or sylvatic foci from non-treated areas, is challenging but essential to evaluate the effectiveness of control initiatives, to identify operational issues regarding suboptimal insecticide application or poor residual activity and to proactively detect incipient or established insecticide resistance.

In this study, we performed high resolution nuclear and mitochondrial genotyping to characterize the dynamics of *T*. *infestans* reinfestation in four rural communities, following an indoor residual spray (IRS) program. To our knowledge this is the first time this phenomenon has been rigorously investigated in the Paraguayan Chaco and interpreted in the context of concomitant human activity [18, 26, 36]. Our understanding of *T*. *infestans* reinfestation in Paraguay is very limited in comparison to neighboring regions in Bolivia and Argentina. While these study data were collected in 2008–2009, strikingly 10 years later, similar scenarios of operational control failure are still reported across the Chaco; although reinfestation processes may share similarities, the Paraguayan Chaco has unique features in terms of housing and sylvatic populations that contribute valuable information to this subject area. Recent studies from Bolivia, indicate more than half of households were found to contain *T*. *infestans* one to two months after alpha-cypermethrin spraying [29]; in this study, household reinfestation in 12J and CL began to rise by three months post-spraying, strongly reinforcing the need to evaluate alternate insecticides and longer-lasting formulations for improved triatomine control in the Gran Chaco. Rapid population recovery has also been demonstrated for pyrethroid susceptible *Triatoma brasiliensis* in Brazil [84] and *T*. *infestans* in Argentina [85], with household infestations returning to baseline levels by 14 months and 2–3 years post-spraying, respectively.

Overall, in our study area, there was a clear absence of isolation by distance and the pattern of genetically distinct populations between close communities were likely caused by local, independent diversification. Considering the genetic profiles of baseline, reinfestant and sylvatic populations in 12J and CL, two distinct scenarios emerge from our analysis. In CL, following spraying, the reinfestant *T*. *infestans* population was likely founded by a small number of domestic individuals from the baseline population which survived insecticidal treatment. While it should be acknowledged, that the small sample size of post-spraying *T*. *infestans* in CL is a study limitation, the *a priori* genetic assignment analysis clearly indicated clustering of pre- and post-spraying populations; furthermore the latter population was characterized by lower numbers of private alleles per locus (0.74 *vs*. 0.44 for pre- and post-spraying, respectively), reduced genetic diversity (0.59 *vs*. 0.18), observed heterozygosity (0.58 *vs*. 0.55; indicative of inbreeding between related individuals), and lower allelic richness (2.78 *vs*. 2.43), compared to both the pre-spraying household population and sylvatic bugs in this area. In addition to colonizing cracks in house walls, triatomines are also known to seek refuge in other household items, including bed frames, mattresses, baskets and clothing; during insecticide application, these possessions are usually removed from the house and replaced after spraying, creating an insecticide-free ‘micro-niche’ within the house for bugs to freely propagate. These *T*. *infestans* populations are then able to migrate between household items and to walls once insecticidal efficacy has waned. Previous studies in the Chaco region indicate that pyrethroid residual efficacy can be low, persisting for three months or less, due to issues of inter- and intra-house dose variability, porous house wall substrates, insecticide degradation induced by high temperatures and dust particles deposited by power sprayers during application, which can block the insecticide [37].

In contrast, the genetic profile of the 12J reinfestant bugs, albeit diverse, were genetically comparable to the pre-spraying population but also to sympatric sylvatic bugs, indicating an admixed origin for this population. Our results suggest that the post-spraying population was seeded by distinct individuals, with different genetic backgrounds, including domestic survivors and invaders from the nearby sylvatic foci. The reinfestation hypothesis for 12J is that domestic populations might not have been fully eliminated (either due to poor residual insecticide efficacy, insufficient chemical application or insecticide resistance) which is reflected in the genetic similarities between baseline and reinfestant 12J populations and lack of reduced diversity post-spraying, expected from genetic bottlenecks imposed by insecticide exposure or invasion from a small founder population. Further support for IRS survivors is the presence of nymphs indoors observed in this community three months post spraying. However, during post-spraying entomological surveys, *T*. *infestans* were captured from peridomestic areas, including chicken coops, where this species was not present during pre-spraying surveillance. Furthermore, low *F*_ST_ values between baseline, reinfestant and sylvatic 12J populations also support a lack of genetic structure in this community, likely indicating gene flow between domestic and sylvatic populations before and after spraying. Remarkably, the genotypes of the few (n = 5) sylvatic triatomines collected in CL–captured more than 10 km away—differed from the closer domestic population characterized in CL, both baseline and reinfestant. However, they were genetically similar to the sylvatic bugs found around 12J, suggesting these genotypes are carried among wild triatomine populations that might be ancient and widespread across the study area. Genetic flow between domestic and sylvatic bugs could occur through mobilization by flying or walking, allowing domestic individuals to settle in the sylvatic area after spraying. These bugs could later reinvade intra- or peridomestic areas after the loss of residual insecticide efficacy. In addition, sylvatic populations have also been shown to mobilize and invade domestic dwellings [29, 79, 80, 86, 87].

Another study from this area detected *T*. *infestans* from 12J which had fed on human blood exclusively (53.3%), as well as on both humans and chickens (46.7%), establishing a link between these domestic and peridomestic transmission cycles [88]. In previous studies, sylvatic *T*. *infestans* from 12J have been collected from up to 2.5 km away from community dwellings and shown to have obtained blood meals from multiple sources, including chickens and humans, further demonstrating the capacity for these populations to mobilize, disperse widely and survive in different microhabitats [26]. Studies from Argentina, Bolivia and Brazil support the hypothesis that triatomine reinfestation dynamics may be strongly associated with features of the peridomestic environment, which constitute suitable artificial ecotopes that provide shelter, blood meal sources and contiguity between domestic and sylvatic environments [29, 84, 89].

While insecticide resistance was not directly tested at the time of this study, no pyrethroid resistance had been reported in *T*. *infestans* populations sampled near to the study area (Elsa López, personal communication). The contrasting post-spraying entomological survey results in 10L and CA, compared to CL and 12J may reflect key differences in insecticide resistance profiles. Populations in both former communities, may be more susceptible to chemical control, as little to no evidence of reinfestation was observed.

Regarding the impact of IRS on parasite populations, *T*. *cruzi* infection prevalence post-spraying was reduced to zero in 10L and CL and to 4.1% (3/73) in 12J (from 11.4%); however, because parasites were detected using microscopy, which is known to have lower sensitivity compared to PCR, prevalence rates may be underestimated in our study. By comparison of three *T*. *infestans* collected from CA post-spraying, two were found infected. Across our study area, TcV and TcVI were the dominant *T*. *cruzi* DTUs found circulating in variable proportions among domestic *T*. *infestans*, with one of their parental lineages, TcII, to a lesser extent. These findings are consistent with previous surveys in the Chaco region where these DTUs are commonly found infecting domestic *T*. *infestans* populations and peridomestic mammals, especially cats and dogs [40, 41, 90–93]. Furthermore, these DTUs have been implicated in severe chronic manifestations of Chagas disease in Southern Cone countries [94–97] representing a significant threat to human health in the Paraguayan Chaco. In Paraguay, TcIII is frequently isolated from the sylvatic environment, particularly from *Dasypus* armadillos [41, 98], while this DTU and TcI are isolated from domestic transmission cycles [99–102] and to date TcIV has not been detected locally. No infected *T*. *sordida* were collected during this study. In a recent work, a domestic specimen of this species was reported infected with *T*. *cruzi*, in Ayoreo Totobiegosode localities (Alto Paraguay-Chaco) [103], although, this low infection rate corroborates the observation that this species is usually associated with bird hosts and may not play an important role in Chagas disease transmission in these communities.

Chagas disease transmission and the operational success of IRS programs are intrinsically linked to house structure and environmental conditions and to the cultural activities of these indigenous groups. The localities of the Enlhet linguistic family (10L, 12J and CL) showed distinct differences in culture and house infestation levels. 10L and 12J both belong to the Angaité ethnic group and were characterized by the lowest housing quality indices, highest triatomine infestation levels, and presence of *T*. *cruzi*, with parasite DTUs that are associated with severe chronic Chagas disease. These localities did not have traditional peridomestic structures to harbor triatomines, and because houses were usually situated immediately adjacent to xerophytic forested areas, apparent gene flow occurred among domestic and sylvatic populations in 12J. Activities of members of the 12J community provide support for the hypothesis that reinfestant *T*. *infestans* populations in 12J immigrated from neighboring unsprayed/sylvatic environments. During the time of this study, community members were constantly mobile; 12J inhabitants were observed frequently dismantling and moving their entire house structures, which were of particularly low quality, and belongings, either to locate water sources within their own territory in times of drought or to observe funeral rites. Community members also maintain their hunter-gatherer customs, frequently leaving to search for food, to care for livestock or to prepare charcoal for sale [104]. These activities occupy community members in the forest for several days, potentially facilitating passive transport of triatomines from the sylvatic environment into the domestic transmission cycle in 12J and *vice versa* [26]. This community has also had extensive exchange with their relatives from the Puerto Casado area, 300 km away, from where they immigrated 30 years ago. Morphometric studies have demonstrated the similarity of wings between *T*. *infestans* populations from 12J and those from Puerto Casado, supporting the hypothesis of passive transport of triatomines between these areas [18]. In contrast, CL house structures were generally better quality than those in 12J with more stable settlements and improved infrastructure and community development. Inhabitants from CL were mostly farmers (74%), with little migration and internal displacement. The locality of CA is composed of the Nivaclé ethnic group, another linguistic family (Mataco Mataguayo), which originated in this study area. The living and educational conditions of this community are higher than the other three localities, as well as the quality of the house construction materials, with agricultural activities employing 58.4% of the population [104]. The initial infestation levels of CA were the lowest of all study communities and households did not experience significant reinfestation post-spraying. The distinct differences in the origins of triatomine populations in CL, CA and 10L, compared to 12J, was further evidenced by bugs from the former communities presenting higher frequencies of mitochondrial haplotype b, the most ubiquitous haplotype of non-Andean *T*. *infestans*, suggesting these populations have been long established in the region.

Aiming to address some of the disparities in economic development and sustainability following this study, during 2012–2013, in 10L and 12J, an innovative social project was carried out to improve house structures, transferring knowledge to local communities regarding alternative materials for construction of dwellings to prevent the establishment of triatomine colonies in walls and roofs, and to adapt the roof for water collection, given the intense drought in the area. The project trained women and men in the construction of dwellings, using soil-cement block construction techniques. Once the construction was finished, a house model was designated as a health post at the request of the community itself. Many young indigenous women were trained in this initiative and went to work outside the community to increase their quality of life; however, very few dwellings were actually improved during this project, because the income obtained from the training was instead used to buy food or other essential supplies for their families [37]. This anecdotal outcome highlights the imperative short-term needs of the local populations that take priority over vector control for Chagas disease, a threat locally perceived as a long-term consequence, and hence addressed with less immediacy.

## Conclusions

The multifactoriality of Chagas disease observed in the studied indigenous communities illustrates the challenges in control and surveillance that National Chagas Disease Programs face. The marked differences in the structure of the dwellings and in the socio-economic level of the inhabitants have not prevented the processes of reinfestation and the dynamics of dispersion of the triatomines between communities. Other factors such as the lack of organization of household items inside the dwellings, which provides safe refuges for triatomine colonies, the cultural migration habits of the communities, which facilitate passive transport of triatomines, as well as the stability of these insect populations in the sylvatic area, their mobility in different environments and the rapid loss of the residual effect of insecticides, reinforce the important roles these events play in the dynamics of *T*. *infestans* persistence and reinfestation in these communities. A thorough knowledge of the ancestral customs of these indigenous groups provided additional insight into the interpretation of study findings. Actions that comprehensively stimulate community development in an integrated manner, together with routine entomological surveillance and timely and effective interventions, are urgently needed to re-address control of Chagas disease transmission in the Paraguayan Chaco.

## Supporting information

S1 File Description of the Gran Chaco linguistic families. (DOCX)

S2 File Cytochrome B sequences used for phylogenetic network analysis. (XLSX)

## References

[1] Gurtler RE, Kitron U, Cecere MC, Segura EL, Cohen JE. Sustainable vector control and management of Chagas disease in the Gran Chaco, Argentina. Proc Natl Acad Sci U S A. 2007;104: 16194–16199. doi: 10.1073/pnas.0700863104 17913895 PMC2042184

[2] Rojas de Arias A, Abad-Franch F, Acosta N, Lopez E, Gonzalez N, Zerba E, et al. Post-control surveillance of *Triatoma infestans* and *Triatoma sordida* with chemically-baited sticky traps. PLoS Negl Trop Dis. 2012;6(9): e1822. doi: 10.1371/journal.pntd.0001822 23029583 PMC3441417

[3] Samuels AM, Clark EH, Galdos-Cardenas G, Wiegand RE, Ferrufino L, Menacho S, et al. Epidemiology of and impact of insecticide spraying on Chagas disease in communities in the Bolivian Chaco. PLoS Negl Trop Dis. 2013;7(8): e2358. doi: 10.1371/journal.pntd.0002358 23936581 PMC3731239

[4] Gurevitz JM, Gaspe MS, Enriquez GF, Provecho YM, Kitron U, Gürtler RE. Intensified surveillance and insecticide-based control of the Chagas disease vector *Triatoma infestans* in the Argentinean Chaco. PLoS Negl Trop Dis. 2013;7: e2158. doi: 10.1371/journal.pntd.0002158 23593525 PMC3623707

[5] Salvatella R, Irabedra P, Castellanos LG. Interruption of vector transmission by native vectors and “the art of the possible”. Mem Inst Oswaldo Cruz. 2014;109: 122–130. doi: 10.1590/0074-0276140338 24626310 PMC4005527

[6] Schmunis G, Zicker F, Moncayo A. Interruption of Chagas’ disease transmission through vector elimination. Lancet. 1996;348: 1171. doi: 10.1016/S0140-6736(05)65305-6 8888192

[7] Dias JC, Schofield.CJ 1998. The control of the transmission by transfusion of Chagas’ disease in the Southern Cone Initiative. Rev Soc Bras Med Trop. 1998;31: 373–383. doi: 10.1590/s0037-86821998000400007 9662965

[8] Hotez PJ. Ten global "hotspots" for the neglected tropical diseases. PLoS Negl Trop Dis. 2014;8: e2496. doi: 10.1371/journal.pntd.0002496 24873825 PMC4038631

[9] Gurevitz JM, Ceballos LA, Gaspe MS, Alvarado-Otegui JA, Enriquez GF, Kitron U, et al. Factors Affecting Infestation by *Triatoma infestans* in a Rural Area of the Humid Chaco in Argentina: A Multi-Model Inference Approach. PLoS Negl Trop Dis. 2011;5: e1349. doi: 10.1371/journal.pntd.0001349 22028941 PMC3196485

[10] Gurtler RE. Sustainability of vector control strategies in the Gran Chaco Region: current challenges and possible approaches. Mem Inst Oswaldo Cruz. 2009;104 Suppl 1: 52–59. doi: 10.1590/s0074-02762009000900009 19753458 PMC3072747

[11] Provecho YM, Gaspe MS, Fernandez MDP, Gurtler RE. House Reinfestation with *Triatoma infestans* (Hemiptera: Reduviidae) after community-wide spraying with insecticides in the Argentine Chaco: a multifactorial process. J Med Entomol. 2017;54: 646–657. doi: 10.1093/jme/tjw224 28399199

[12] Schofield CJ, Jannin J, Salvatella R. The future of Chagas disease control. Trends Parasitol. 2006;22: 583–588. doi: 10.1016/j.pt.2006.09.011 17049308

[13] Noireau F, Cortez MG, Monteiro FA, Jansen AM, Torrico F. Can wild *Triatoma infestans* foci in Bolivia jeopardize Chagas disease control efforts? Trends Parasitol. 2005;21: 7–10. doi: 10.1016/j.pt.2004.10.007 15639733

[14] Guhl F, Pinto N, Aguilera G. Sylvatic triatominae: a new challenge in vector control transmission. Mem Inst Oswaldo Cruz. 2009;104: 71–75. doi: 10.1590/s0074-02762009000900012 19753461

[15] Sanchez-Martin MJ, Feliciangeli MD, Campbell-Lendrum D, Davies CR. Could the Chagas disease elimination programme in Venezuela be compromised by reinvasion of houses by sylvatic *Rhodnius prolixus* bug populations? Trop Med Int Health. 2009;11: 1585–1593. 10.1111/j.1365-3156.2006.01717.x 17002733

[16] World Health Organization. 2010. Chagas disease: control and elimination. Report by the Secretariat. Sixty-Third World Health Assembly Provisional Agenda Item 11.14. A63/17.

[17] World Health Organization. Chagas disease in Latin America: an epidemiological update based on 2010 estimates. Wkly Epidemiol Rec. 2015;90: 33–44. 25671846

[18] Rojas de Arias AR, Carbajal de la Fuente AL, Gómez A, Cecere MC, Rolón M, Gómez MCV, et al. Morphometric Wings Similarity among Sylvatic and Domestic Populations of *Triatoma infestans* (Hemiptera: Reduviidae) from the Gran Chaco Region of Paraguay. Am J Trop Med Hyg. 2017;97(2): 481–488. doi: 10.4269/ajtmh.16-1013 28829725 PMC5544089

[19] Ceballos LA, Piccinali RV, Berkunsky I, Kitron U, Gurtler RE. First finding of melanic sylvatic *Triatoma infestans* (Hemiptera: Reduviidae) colonies in the Argentine Chaco. J Med Entomol. 2009;46: 1195–1202. doi: 10.1603/033.046.0530 19769054 PMC2782367

[20] Noireau F, Bastrenta B, Catala S, Dujardin JP, Panzera F, Torres M, et al. Sylvatic population of *Triatoma infestans* from the Bolivian Chaco: from field collection to characterization. Mem Inst Oswaldo Cruz. 2000;95 Suppl 1: 119–122. doi: 10.1590/s0074-02762000000700020 11142700

[21] Noireau F, Flores R, Gutierrez T, Dujardin JP. Detection of sylvatic dark morphs of *Triatoma infestans* in the Bolivian Chaco. Mem Inst Oswaldo Cruz. 1997;92: 583–584. doi: 10.1590/s0074-02761997000500003 9566222

[22] Buitrago R, Waleckx E, Bosseno MF, Zoveda F, Vidaurre P, Salas R, et al. E. First report of widespread wild populations of *Triatoma infestans* (Reduviidae, Triatominae) in the valleys of La Paz, Bolivia. Am J Trop Med Hyg. 2010;82: 574–579. doi: 10.4269/ajtmh.2010.09-0325 20348501 PMC2844558

[23] Waleckx E, Depickere S, Salas R, Aliaga C, Monje M, Calle H, et al. New discoveries of sylvatic *Triatoma infestans* (Hemiptera: Reduviidae) throughout the Bolivian Chaco. Am J Trop Med Hyg. 2012;86: 455–458. doi: 10.4269/ajtmh.2012.11-0205 22403316 PMC3284361

[24] Bacigalupo A, Segura JA, Garcia A, Hidalgo J, Galuppo S, Cattan PE. First finding of Chagas disease vectors associated with wild bushes in the Metropolitan Region of Chile. Rev Med Chil. 2006;134: 1230–1236. doi: 10.4067/s0034-98872006001000003 17186091

[25] Bacigalupo A, Torres-Perez F, Segovia V, Garcia A, Correa JP, Moreno L, et al. 2010. Sylvatic foci of the Chagas disease vector *Triatoma infestans* in Chile: description of a new focus and challenges for control programs. Mem Inst Oswaldo Cruz. 2010;105: 633–641. doi: 10.1590/s0074-02762010000500006 20835609

[26] Rolón M, Vega MC, Román F, Gómez A, Rojas de Arias A. First report of colonies of sylvatic *Triatoma infestans* (Hemiptera: Reduviidae) in the Paraguayan Chaco, using a trained dog. PLoS Negl Trop Dis. 2011;5: e1026. doi: 10.1371/journal.pntd.0001026 21572522 PMC3086807

[27] Breniere SF, Salas R, Buitrago R, Bremond P, Sosa V, Bosseno MF, et al. Wild populations of *Triatoma infestans* are highly connected to intra-peridomestic conspecific populations in the Bolivian Andes. PLoS One. 2013;8: e80786. doi: 10.1371/journal.pone.0080786 24278320 PMC3835561

[28] Bremond P, Salas R, Waleckx E, Buitrago R, Aliaga C, Barnabe C, et al. Variations in time and space of an Andean wild population of *T*. *infestans* at a microgeographic scale. Parasit Vectors. 2014;7: 164. doi: 10.1186/1756-3305-7-164 24708673 PMC3992151

[29] Perez-Cascales E, Sossa-Soruco VM, Breniere SF, Depickere S. Reinfestation with *Triatoma infestans* despite vigilance efforts in the municipality of Saipina, Santa Cruz, Bolivia: situational description two months after fumigation. Acta Trop. 2020;203: 105292. doi: 10.1016/j.actatropica.2019.105292 31816321

[30] Piccinali RV, Fronza G, Mougabure-Cueto GA, Toloza AC. Genetic structure of deltamethrin-resistant populations of *Triatoma infestans* (Hemiptera: Reduviidae) in the Gran Chaco. Parasitol Res. 2020;119(10): 3305–3313. doi: 10.1007/s00436-020-06789-y 32651636

[31] Marcet PL, Santo-Orihuela P, Messenger LA, Vassena CV. Genetic structure and sodium channel gene mutation analysis related to pyrethroid insecticide toxicity in sylvatic Andean *Triatoma infestans* from Bolivia. bioRxiv 512392.

[32] Santo-Orihuela PL, Carvajal G, Picollo MI, Vassena CV. Toxicological and biochemical analysis of the susceptibility of sylvatic *Triatoma infestans* from the Andean Valley of Bolivia to organophosphate insecticide. Mem Inst Oswaldo Cruz. 2013;108: 790–795. doi: 10.1590/0074-0276108062013017 24037203 PMC3970684

[33] Echeverria JE, Bustamante Gomez MB, D’Avila Pessoa GC, Cortez MR, Rodriguez AN, Diotaiuti LG. Resistance to deltamethrin by domestic and wild *Triatoma infestans* populations in the municipality of Toro Toro, Potosi, Bolivia. Parasit Vectors. 2018;11: 92. doi: 10.1186/s13071-018-2663-5 29454379 PMC5816527

[34] Fabre A. Los enlhet-enenlhet del Chaco paraguayo. Pages 503–569 Suplemento Antropológico, Asunción. 2005.

[35] Canese J, Brice E. Elevado índice de serología positiva para la enfermedad de Chagas en el Chaco Paraguayo. XV Departamento Presidente Hayes. Rev Paraguaya Microbio/13(1)3–6, 1978.

[36] Arrom-Suhurt CM, Arrom-Suhurt CH, Arrom-Suhurt MA, Rolon M, Vega-Gomez MC, Rojas de Arias A. Socioeconomic profile and perceptions of Chagas disease in indigenous communities of the Paraguayan Chaco. Journal of Public Health. 2019;27: 723–732.

[37] Rojas de Arias A, Lehane MJ, Schofield CJ, Maldonado M. Pyrethroid insecticide evaluation on different house structures in a Chagas disease endemic area of the Paraguayan Chaco. Mem Inst Oswaldo Cruz. 2004;99(6): 657–62. doi: 10.1590/s0074-02762004000600022 15558181

[38] Rojas de Arias A. 2016. La Certificación del Corte de Transmisión Vectorial del *Trypanosoma cruzi*, agente etiológico de la enfermedad de Chagas. Mem Inst Investig Cienc Salud. 2016;14(3): 3–6.

[39] Olmos F, Mario S, Gomez A. Hábitat y vivienda por medio de datos censales. Calidad de los materiales (CALMAT). Argentina: DNESyP/DEP/P5/PID; 2003. Serie Hábitat y Vivienda DT N° 13.

[40] Acosta N, López E, Lewis MD, Llewellyn MS, Gómez A, Román F, et al. Hosts and vectors of *Trypanosoma cruzi* discrete typing units in the Chagas disease endemic region of the Paraguayan Chaco. Parasitology. 2017;144(7): 884–898. doi: 10.1017/S0031182016002663 28179034 PMC5471830

[41] Yeo M, Acosta N, Llewellyn MS, Sanchez H, Adamson S, Miles GAJ, et al. Origins of Chagas disease: Didelphis species are natural hosts of *Trypanosoma cruzi* I and armadillo hosts of *Trypanosoma cruzi* II, including hybrids. Int J Parasitol. 2005;35(2): 225–33. doi: 10.1016/j.ijpara.2004.10.024 15710443

[42] Lewis MD, Ma J, Yeo M, Carrasco HJ, Llewellyn MS, Miles MD. Genotyping of *Trypanosoma cruzi*: systematic selection of assays allowing rapid and accurate discrimination of all known lineages. Am J Trop Med Hyg. 2009;81(6): 1041–9. doi: 10.4269/ajtmh.2009.09-0305 19996435 PMC2825677

[43] Souto RP, Fernandes O, Macedo AM, Campbell, Zingales B. DNA markers define two major phylogenetic lineages of *Trypanosoma cruzi*. Mol Biochem Parasitol. 1996;83: 141–152. doi: 10.1016/s0166-6851(96)02755-7 9027747

[44] Brisse S, Dujardin JC, Tibayrenc M. Identification of six *Trypanosoma cruzi* lineages by sequence-characterised amplified region markers. Mol Biochem Parasitol. 2000;111: 95–105. doi: 10.1016/s0166-6851(00)00302-9 11087920

[45] Garcia AL, Carrasco HJ, Schofield CJ, Stothard JR, Frame IA, Valente SAS, et al. Random amplification of polymorphic DNA as a tool for taxonomic studies of triatomine bugs (Hemiptera: Reduviidae). J Med Entomol. 1998;35: 38–45. doi: 10.1093/jmedent/35.1.38 9542343

[46] Breniere SF, Bosseno MF, Telleria J, Carrasco R, Vargas F, Yaksic N, et al. Field application of polymerase chain reaction diagnosis and strain typing of *Trypanosoma cruzi* in Bolivian triatomines. Am J Trop Med Hyg. 1995;53: 179–184. doi: 10.4269/ajtmh.1995.53.179 7677221

[47] Marcet PL, Lehmann T, Groner G, Gürtler RE, Kitron U, Dotson EM. Identification and characterization of microsatellite markers in the Chagas disease vector *Triatoma infestans* (Heteroptera: Reduviidae). Infect Genet Evol. 2006;6: 32–37. doi: 10.1016/j.meegid.2005.01.002 16376838 PMC1351232

[48] Marcet PL, Mora MS, Cutrera AP, Jones L, Gurtler RE, Kitron U, et al. Genetic structure of *Triatoma infestans* populations in rural communities of Santiago Del Estero, northern Argentina. Infect Genet Evol. 2008;8: 835–846. doi: 10.1016/j.meegid.2008.08.002 18773972 PMC3680132

[49] Weir BS, Cockerham CC. Estimating F-statistics for the analysis of population structure. Evolution. 1984;38: 1358–1370. doi: 10.1111/j.1558-5646.1984.tb05657.x 28563791

[50] El Mousadik A, Petit R. High level of genetic differentiation for allelic richness among populations of the argan tree (*Argania spinosa* (L.) Skeels) endemic to Morocco. Theor Appl Genet. 1996;92: 832–839. doi: 10.1007/BF00221895 24166548

[51] Nei M. Molecular Evolutionary Genetics. Columbia University Press, New York. 1987.

[52] Goudet J. FSTAT version 1.2: a computer program to calculate F-statistics Journal of Heredity. 1995;86: 485–486.

[53] Rice W. Analyzing tables of statistical tests. Evolution. 1989;43: 223–225. doi: 10.1111/j.1558-5646.1989.tb04220.x 28568501

[54] Kalinowski ST. HP-Rare: A Computer Program for Performing Rarefaction on Measures of Allelic Diversity. Mol Ecol Notes. 2005;5: 187–189.

[55] Peakall R, Smouse PE. GenAlEx 6.5: genetic analysis in Excel. Population genetic software for teaching and research—an update. Bioinformatics. 2012;28: 2537–2539. doi: 10.1093/bioinformatics/bts460 22820204 PMC3463245

[56] Falush D, Stephens M, Pritchard JK. Inference of population structure using multilocus genotype data: dominant markers and null alleles. Mol Ecol Notes. 2007;7(4): 574–578. doi: 10.1111/j.1471-8286.2007.01758.x 18784791 PMC1974779

[57] Hubisz MJ, Falush D, Stephens M, Pritchard JK. Inferring weak population structure with the assistance of sample group information. Mol Ecol Resour. 2009;9: 1322–1332. doi: 10.1111/j.1755-0998.2009.02591.x 21564903 PMC3518025

[58] Pritchard JK, Stephens M, Donnelly P. Inference of population structure using multilocus genotype data. Genetics. 2000;155(2): 945–959. doi: 10.1093/genetics/155.2.945 10835412 PMC1461096

[59] Evanno G, Regnaut S, Goudet J. Detecting the number of clusters of individuals using the software STRUCTURE: a simulation study. Mol Ecol. 2005;14: 2611–2620. doi: 10.1111/j.1365-294X.2005.02553.x 15969739

[60] Earl DA, vonHoldt BM. STRUCTURE HARVESTER: a website and program for visualizing STRUCTURE output and implementing the Evanno method. Conservation Genetics Resources. 2012;4: 359–361.

[61] Lyman DF, Monteiro FA, Escalante AA, Cordon-Rosales C, Wesson DM, Dujardin JP, et al. Mitochondrial DNA sequence variation among triatomine vectors of Chagas’ disease. Am J Trop Med Hyg. 1999;60: 377–386. doi: 10.4269/ajtmh.1999.60.377 10466963

[62] Hall TA. BioEdit: a user-friendly biological sequence alignment editor and analysis program for Windows 95/98/NT. Nucl Acids Symp. 1999;Ser. 41: 95–98.

[63] Librado P, Rozas J. DnaSP v5: a software for comprehensive analysis of DNA polymorphism data. Bioinformatics. 2009;25: 1451–1452. doi: 10.1093/bioinformatics/btp187 19346325

[64] Bandelt HJ, Forster P, Rohl A. Median-joining networks for inferring intraspecific phylogenies. Mol Biol Evol. 1999;16: 37–48. doi: 10.1093/oxfordjournals.molbev.a026036 10331250

[65] Leigh JW, Bryant D. popart: full-feature software for haplotype network construction. Methods Ecol Evol. 2015;6: 1110–1116.

[66] Botstein D, White RL, Skolnick M, Davis RW. Construction of a genetic linkage map in man using restriction fragment length polymorphisms. Am J Hum Genet. 1980;32: 314–331. 6247908 PMC1686077

[67] Falush D, Stephens M, Pritchard JK. Inference of population structure using multilocus genotype data: linked loci and correlated allele frequencies. Genetics. 2003;164: 1567–1587. doi: 10.1093/genetics/164.4.1567 12930761 PMC1462648

[68] Monteiro FA, Perez R, Panzera F, Dujardin JP, Galvao C, Rocha D, et al. Mitochondrial DNA variation of *Triatoma infestans* populations and its implication on the specific status of *T*. *melanosoma*. Mem Inst Oswaldo Cruz. 1999;94 Suppl 1: 229–238. doi: 10.1590/s0074-02761999000700037 10677723

[69] Ceballos LA, Piccinali RV, Marcet PL, Vazquez-Prokopec GM, Cardinal MV, Schachter-Broide J, et al. Hidden Sylvatic Foci of the Main Vector of Chagas Disease *Triatoma infestans*: Threats to the Vector Elimination Campaign? PLoS Negl Trop Dis. 2011;5: e1365. doi: 10.1371/journal.pntd.0001365 22039559 PMC3201917

[70] Justi SA, Russo CA, Mallet JR, Obara MT, Galvao C. Molecular phylogeny of Triatomini (Hemiptera: Reduviidae: Triatominae). Parasit Vectors. 2014;7(1): 149. 24685273 10.1186/1756-3305-7-149 PMC4021723

[71] Barnabe C, Grijalva MJ, Santillan-Guayasamin S, Yumiseva CA, Waleckx E, Breniere SF, et al. Genetic data support speciation between *Panstrongylus howardi* and *Panstrongylus chinai*, vectors of Chagas disease in Ecuador. Infect Genet Evol. 2020;78: 104103. doi: 10.1016/j.meegid.2019.104103 31698115

[72] Giordano R, Pizarro JC, Paulk S, Stevens L. Genetic diversity of *Triatoma infestans* (Hemiptera: Reduviidae) in Chuquisaca, Bolivia based on the mitochondrial cytochrome b gene. Mem Inst Oswaldo Cruz. 2005;100(7): 753–760. doi: 10.1590/s0074-02762005000700014 16410965

[73] Quisberth S, Waleckx E, Monje M, Chang B, Noireau F, Brenière SF. "Andean" and "non-Andean" ITS-2 and mtCytB haplotypes of *Triatoma infestans* are observed in the Gran Chaco (Bolivia): Population genetics and the origin of reinfestation. Infect Genet Evol. 2011;11: 1006–1014. doi: 10.1016/j.meegid.2011.03.014 21457795

[74] Waleckx E, Salas R, Huamán N, Buitrago R, Bosseno M-F, Aliaga C, et al. 2011. New insights on the Chagas disease main vector *Triatoma infestans* (Reduviidae, Triatominae) brought by the genetic analysis of Bolivian sylvatic populations. Infect Genet Evol. 2011;11: 1045–1057. doi: 10.1016/j.meegid.2011.03.020 21463708

[75] Bern C. Chagas’ Disease. N Engl J Med. 2015;373(5): 456–66. doi: 10.1056/NEJMra1410150 26222561

[76] Rassi A Jr., Rassi A, Marin-Neto JA. Chagas disease. Lancet. 2010;375(9723): 1388–402. doi: 10.1016/S0140-6736(10)60061-X 20399979

[77] Gaspe MS, Provecho YM, Cardinal MV, del Pilar Fernandez M, Gurtler RE. Ecological and sociodemographic determinants of house infestation by *Triatoma infestans* in indigenous communities of the Argentine Chaco. PLoS Negl Trop Dis. 2015;9(3): e0003614. doi: 10.1371/journal.pntd.0003614 25785439 PMC4364707

[78] Cardinal MV, Orozco MM, Enriquez GF, Ceballos LA, Gaspe MS, Alvarado-Otegui JA, et al. Heterogeneities in the ecoepidemiology of *Trypanosoma cruzi* infection in rural communities of the Argentinean Chaco. Am J Trop Med Hyg. 2014;90(6): 1063–1073. doi: 10.4269/ajtmh.13-0251 24732461 PMC4047730

[79] Vazquez-Prokopec GM, Ceballos LA, Marcet PL, Cecere MC, Cardinal MV, Kitron U, et al. Seasonal variations in active dispersal of natural populations of *Triatoma infestans* in rural north-western Argentina. Med Vet Entomol. 2006;20: 1–6. doi: 10.1111/j.1365-2915.2006.00614.x 17044877 PMC1894892

[80] Abrahan LB, Goria DA, Catala SS. Dispersal of *Triatoma infestans* and other Triatominae species in the arid Chaco of Argentina: flying, walking or passive carriage? The importance of walking females. Mem Inst Oswaldo Cruz. 2011;106: 232–239. doi: 10.1590/s0074-02762011000200019 21537686

[81] Cecere MC, Vazquez-Prokopec GM, Ceballos LA, Boragno S, Zarate JE, Kitron U, et al. Improved chemical control of Chagas disease vectors in the dry Chaco region. J Med Entomol. 2013;50(2): 394–403. doi: 10.1603/me12109 23540129 PMC3773707

[82] Gomez MB, Diotaiuti LG, Gorla DE. et al. Distribution of pyrethroid resistant populations of *Triatoma infestans* in the southern cone of South America. PLoS Negl Trop Dis. 2016;10(3): e0004561. doi: 10.1371/journal.pntd.0004561 27007658 PMC4805280

[83] Gurevitz JM, Gaspe MS, Enriquez GF, Vassena CV, Alvarado-Otegui J, Provecho YM, et al. Unexpected failures to control Chagas disease vectors with pyrethroid spraying in northern Argentina. J Med Entomol. 2012;49(6): 1379–1386. doi: 10.1603/me11157 23270166 PMC3760256

[84] Bezerra CM, Barbosa SE, de Souza R de CM, Feijao LX, Gurtler RE, Ramos AN Jr., et al. L. Fast recovery of house infestation with *Triatoma brasiliensis* after residual insecticide spraying in a semiarid region of Northeastern Brazil. PLoS Negl Trop Dis. 2020;14(7): e0008404. doi: 10.1371/journal.pntd.0008404 32687497 PMC7371158

[85] Gurtler RE, Cecere MC, Lauricella MA, Petersen RM, Chuit R, Segura EL, et al. Incidence of *Trypanosoma cruzi* infection among children following domestic reinfestation after insecticide spraying in rural northwestern Argentina. Am J Trop Med Hyg. 2005;73: 95–103. 16014842 PMC1351233

[86] Abrahan L, Gorla D, Catala S. Active dispersal of *Triatoma infestans* and other triatomines in the Argentinean arid Chaco before and after vector control interventions. J Vect Ecol. 2016;41(1): 90–96. 10.1111/jvec.12198 27232129

[87] Almeida CE, Faucher L, Lavina M, Costa J, Harry M. Molecular individual-based approach on *Triatoma brasiliensis*: inferences on triatomine foci, *Trypanosoma cruzi* natural infection prevalence, parasite diversity and feeding sources. PLoS Negl Trop Dis. 2016;10(2): e0004447. doi: 10.1371/journal.pntd.0004447 26891047 PMC4758651

[88] Fraenkel S, Salvioni OD, Rojas de Arias A, Arze VP, Rolon M, Ramirez N, et al. Identification of bloodmeal sources of triatomines captured in the Paraguayan Chaco region of South America by means of molecular biology analysis. Pathog Glob Health. 2020;114: 30–39. doi: 10.1080/20477724.2020.1716558 31973639 PMC7144232

[89] Cecere MC, Vazquez-Prokopec G, Gürtler RE, Kitron U. Reinfestation Sources for Chagas Disease Vector, Triatoma infestans, Argentina. Emerg Infect Dis. 2006;12(7):1096–1102. doi: 10.3201/eid1207.051445 16836826 PMC1853288

[90] Enriquez GF, Cardinal MV, Orozco MM, Lanati L, Schijman AG, Gurtler RE. Discrete typing units of *Trypanosoma cruzi* identified in rural dogs and cats in the humid Argentinean Chaco. Parasitology. 2013;140: 303–8. doi: 10.1017/S003118201200159X 23058180 PMC3721149

[91] Perez E, Monje M, Chang B, Buitrago R, Parrado R, Barnabe C, et al. Predominance of hybrid discrete typing units of *Trypanosoma cruzi* in domestic *Triatoma infestans* from the Bolivian Gran Chaco region. Infect Genet Evol. 2013;13: 116–23. doi: 10.1016/j.meegid.2012.09.014 23047136

[92] Fernández M del P, Cecere MC, Lanati LA, Lauricella MA, Schijman AG, Gürtler RE, et al. Geographic variation of *Trypanosoma cruzi* discrete typing units from *Triatoma infestans* at different spatial scales. Acta Trop. 2014;140: 10–18. doi: 10.1016/j.actatropica.2014.07.014 25090650

[93] Macchiaverna NP, Enriquez GF, Buscaglia CA, Balouz V, Gurtler RE, Cardinal MV. New human isolates of *Trypanosoma cruzi* confirm the predominance of hybrid lineages in domestic transmission cycle of the Argentinean Chaco. Infect Genet Evol. 2018;66: 229–235. doi: 10.1016/j.meegid.2018.10.001 30296602

[94] Cura CI, Lucero RH, Bisio M, Oshiro E, Formichelli LB, Burgos JM, et al. *Trypanosoma cruzi* discrete typing units in Chagas disease patients from endemic and non-endemic regions of Argentina. Parasitology. 2012;139: 516–521. doi: 10.1017/S0031182011002186 22309735

[95] Vicco MH, Bontempi I, Ortiz S, Solari A, Bottasso OA Marcipar I. Chronic Chagas disease with cardiodigestive involvement and the TcVI infective form of *Trypanosoma cruzi*. A case report. Parasitol Int. 2012;61: 735–737. doi: 10.1016/j.parint.2012.04.007 22564509

[96] Messenger LA, Miles MA, Bern C. Between a bug and a hard place: *Trypanosoma cruzi* genetic diversity and the clinical outcomes of Chagas disease. Expert Rev Anti Infect Ther. 2015;13(8): 995–1029. doi: 10.1586/14787210.2015.1056158 26162928 PMC4784490

[97] Lucero RH, Brusés BL, Cura CI, Formichelli LB, Juiz N, Fernández GJ, et al. Chagas’ disease in Aboriginal and Creole communities from the Gran Chaco Region of Argentina: seroprevalence and molecular parasitological characterization. Infect Genet Evol. 2016;41: 84–92. doi: 10.1016/j.meegid.2016.03.028 27057620

[98] Llewellyn MS, Lewis MD, Acosta N, Yeo M, Carrasco HJ, Segovia M, et al. *Trypanosoma cruzi* IIc: phylogenetic and phylogeographic insights from sequence and microsatellite analysis and potential impact on emergent Chagas disease. PLoS Negl Trop Dis. 2009;3: e510. doi: 10.1371/journal.pntd.0000510 19721699 PMC2727949

[99] Cura CI, Mejía-Jaramillo AM, Duffy T, Burgos JM, Rodriguero M, Cardinal MV, et al. *Trypanosoma cruzi* I genotypes in different geographical regions and transmission cycles based on a microsatellite motif of the intergenic spacer of spliced-leader genes. Int J Parasitol. 2010;40: 1599–1607. doi: 10.1016/j.ijpara.2010.06.006 20670628 PMC3081674

[100] Risso M.G, Sartor PA, Burgos JM, Briceño L, Rodríguez EM, Guhl F, et al. (2011). Immunological identification of *Trypanosoma cruzi* lineages in human infection along the endemic area. Am J Trop Med Hyg. 2011;84: 78–84. doi: 10.4269/ajtmh.2011.10-0177 21212206 PMC3005503

[101] Chapman MD, Baggaley RC, Godfrey-Fausset PF, Malpas TJ, White G, Canese J. et al. *Trypanosoma cruzi* from the Paraguayan Chaco: isoenzyme profiles of strains isolated at Makthlawaiya. J Protozool. 1984;31: 482–486. doi: 10.1111/j.1550-7408.1984.tb02999.x 6239030

[102] Sánchez Z, Russomando G, Chena L, Nara E, Cardozo E, Paredes B et al. *Triatoma sordida* indicadores de adaptación y transmisión de *Trypanosoma cruzi* en intradomicilio del Chaco Paraguayo. Mem Inst Investig Cienc Salud. 2016;14(3): 96–101.

[103] Sanchez Casaccia P, Gonzalez-Britez N, Acosta N, Lopez E. Vectores de *Trypanosoma cruzi* en ambientes domesticos y silvestres de las comunidades Ayoreo Totobiegosode del Alto Paraguay. Rev Soc Cient Parag. 2019;24(1): 218–229.

[104] DGEEC. Anuario Estadistico del Paraguay 2002.

