# Supplementary material for: Dynamics of Triatoma infestans populations in the Paraguayan Chaco: Population genetic analysis of household reinfestation following vector control
Source: PLoS One. 2022 Feb 10;17(2):e0263465. doi: 10.1371/journal.pone.0263465 (PMC8830694; doi:10.1371/journal.pone.0263465)
Supplement: S1 File — (DOCX) [file pone.0263465.s001.docx]

**Description of the Gran Chaco Linguistic Families**

The Gran Chaco, which extends into Bolivia, Argentina and Paraguay, is hyper-endemic for Chagas disease and the heart of the current problem of Chagas disease. Despite an on-going elimination campaign carried out at regional levels through the "Southern Cone Initiative", transmission mediated by the vector *Triatoma infestans* persists in much of the Gran Chaco, with prevalence ranging from 1 to 20% of households infested and 20% of the population infected.

In the Chaco, there are 20 linguistic families: the Ayoreo, in the north and northeast of the Chaco, belonging to the Zamuco linguistic family; in the central-western part, the Maskoy linguistic family, with the Sanapaná, Enlhet, Angaité and Lengua ethnic groups; from the centre of the Chaco to the Pilcomayo River, the Nivaclé or Chulupíes, of the Mataco Mataguayo linguistic family; and finally, the Tupí Guaraní, with the Guaraní Ñandeva and Guarayos ethnic groups. These indigenous groups were hunter-gatherers and horticulturists; however, since the creation of the tanneries and the Mennonite colonies, they have worked through *changas* or salaried jobs as a source of primary subsistence. The linguistic heterogeneity, the lack of global knowledge and the lack of organization of many of these groups are obvious obstacles to carrying out Chagas disease information and education campaigns.

The illiteracy rate among indigenous people is extremely high, averaging 51%, a rate far higher than that of the Paraguayan population in general, which is only 7.1%. According to the National Indigenous Census, an average of 42.8% of indigenous women never attended school and 35.8% of men did not. These values are much higher than those for the country's rural population in general, which are 10.9% and 8.7% for women and men, respectively.

In most communities, houses have a zinc roof to alleviate the problem of water access (by enabling water collection), a vital resource whose scarcity in the region makes these communities even more vulnerable. The Nivaclé and Angaité groups predominantly use tin roofs (mud and straw). In the interior of the house, almost always in one place, one can see an enormous accumulation of household items. These items are taken out during chemical spraying of the house, however triatomines (of all life cycle stages) can persist in these items, making it very difficult to eliminate them entirely. These communities have very few peridomestic areas, instead we observe a predominance of kitchens outdoors, with fires directly on the ground, in addition to precarious or simply non-existent bathrooms. The average number of inhabitants in this type of housing is 5 or more, which shows a significant level of overcrowding. As can be seen from the type of walls, the accumulation of belongings and overcrowding inside the houses in all groups, facilitates the proliferation of vectors of Chagas disease.

The people belonging to the Maskoy linguistic family are characterized as nomadic hunters, inscribed within the Palaeolithic socio-cultural pattern of intensive use of "all subsistence, natural or circumstantial resources". The immediate needs and their dependence on natural resources that are increasingly difficult to acquire due to their displacement from their original lands and consequently the loss of their free mobility, together with the "utilitarian pragmatism that guides the socioeconomic adaptation of the small communities", led to a process of adaptation to the new reality and to the working patterns in the coexistence with the white (mostly Mennonite) environment.

**Social and Geographical Vulnerabilities**

The Paraguayan Chaco represents 60% of the national territory. Approximately 264,000km^2^, in which only 3% of the Paraguayan population lives, is distributed among indigenous groups, Mennonite colonies and Creole/Latino people.

The indigenous population is clearly the most vulnerable group of people, suffering from a range of problems, including:

- Conditions of exposure, fragility and resilience

- Physical, institutional and political isolation

- Lack of water sources and availability of safe water supplies

- Difficulties in accessing traditional foods and markets

- Poverty and low coverage of basic services and needs (roads, health posts, schools, drinking water, electricity, and communication)

- Low community organization and loss of cultural identity (lack of self-determination)

- Marginalization and socio-cultural conflicts with national authorities and institutions

- Weak presence of the State

**Alteration of the Territory**

The alteration of the territory increased by climatic pressure variability / change and anthropic economic-productive processes (intensive livestock rearing) that degrade the balance of the environment and exacerbate socio-cultural conflicts, with some minorities of owners and large ranchers who maintain the monopoly of existing resources, while indigenous groups struggle for subsistence on a daily basis that keeps them below the poverty line. There is also a glaring absence of the State, so these indigenous populations lack public institutions to support their management. Since 2008, the indigenous groups have received support for the construction of housing, which has not yet materialized, and they receive regular visits from the family health units of the Ministry of Health.

**Study Area**

The study area is 557km from Asunción, the capital city. The communities that surround the area are:

**Ethnic Nivaclé of the Campo Alegre Village**

This group is located in a rural area of the Department of Boquerón, in the district of Mcal. José F. Estigarribia, rural area, 62km from the departmental capital (Philadelphia) and 125km from the district capital, (Mcal. José F. Estigarribia). Belonging to the linguistic family Mataco Mataguayo, the language spoken in the community is Nivaclé, (98%); with small proportions of individuals who speak Spanish (1.3%) and Guaraní, (0.4%). This ethnic group has legal status. It is composed of 73 families and the average number of years of study was 2.9 in 2002.

**Enlhet ethnic group North of the Campo Largo Village**

This group is located in the Department of President Hayes, in the district of Villa Hayes, rural area. It is 446km away from the capital city of the department (Villa Hayes) and from the district capital. Belonging to the linguistic family Maskoy language, the language spoken is the northern Enlhet (99.4%); a small proportion of individuals also speak Nivaclé (0.6%). It has approximately 110 families with an average of 4.6 inhabitants per house. The average number of years of study was 2.5 in 2002.

**Angaité ethnic group of the 10 Leguas Village**

This group is located in the Department of President Hayes, in the district of Villa Hayes, in a rural area. It is located 427km from the capital of the department (Villa Hayes) and from the district capital. This ethnic group belongs to the Maskoy language family, the spoken language is Guarani (100%), and they have legal status and their own land. There are 34 families and the average number of inhabitants per house is 6.4, the average number of years of study was 1.0 in 2002. It is 8km from 12 June.

**Angaité ethnic group of the 12 de Junio Village**

This group is located in the Department of President Hayes, in the district of Benjamin Aceval, rural area, 402km away from the capital of the department (Villa Hayes) and the district capital (Benjamín Aceval). It belongs to the Maskoy language family. The language spoken is Guarani. There are currently 96 families and the average number of years of study was 0.6 in 2002.
